# Exome Sequencing of Cell-Free DNA from Metastatic Cancer Patients Identifies Clinically Actionable Mutations Distinct from Primary Disease

**DOI:** 10.1371/journal.pone.0136407

**Published:** 2015-08-28

**Authors:** Timothy M. Butler, Katherine Johnson-Camacho, Myron Peto, Nicholas J. Wang, Tara A. Macey, James E. Korkola, Theresa M. Koppie, Christopher L. Corless, Joe W. Gray, Paul T. Spellman

**Affiliations:** Knight Cancer Institute, Oregon Health and Sciences University, Portland, Oregon, United States of America; Cornell University, UNITED STATES

## Abstract

The identification of the molecular drivers of cancer by sequencing is the backbone of precision medicine and the basis of personalized therapy; however, biopsies of primary tumors provide only a snapshot of the evolution of the disease and may miss potential therapeutic targets, especially in the metastatic setting. A liquid biopsy, in the form of cell-free DNA (cfDNA) sequencing, has the potential to capture the inter- and intra-tumoral heterogeneity present in metastatic disease, and, through serial blood draws, track the evolution of the tumor genome.

In order to determine the clinical utility of cfDNA sequencing we performed whole-exome sequencing on cfDNA and tumor DNA from two patients with metastatic disease; only minor modifications to our sequencing and analysis pipelines were required for sequencing and mutation calling of cfDNA. The first patient had metastatic sarcoma and 47 of 48 mutations present in the primary tumor were also found in the cell-free DNA. The second patient had metastatic breast cancer and sequencing identified an *ESR1* mutation in the cfDNA and metastatic site, but not in the primary tumor. This likely explains tumor progression on Anastrozole. Significant heterogeneity between the primary and metastatic tumors, with cfDNA reflecting the metastases, suggested separation from the primary lesion early in tumor evolution. This is best illustrated by an activating *PIK3CA* mutation (H1047R) which was clonal in the primary tumor, but completely absent from either the metastasis or cfDNA. Here we show that cfDNA sequencing supplies clinically actionable information with minimal risks compared to metastatic biopsies. This study demonstrates the utility of whole-exome sequencing of cell-free DNA from patients with metastatic disease. cfDNA sequencing identified an *ESR1* mutation, potentially explaining a patient’s resistance to aromatase inhibition, and gave insight into how metastatic lesions differ from the primary tumor.

## Introduction

In 2014 there were be over 500,000 cancer related deaths in the United States; 90% of these deaths from metastatic disease.[1, 2] While cancer is characterized by clonal progression, metastatic lesions and recurrent disease can differ substantially from the primary tumor, harboring unique mutations of clinical significance.[3] Identifying these differences as they emerge requires serial sampling of the tumor genome,[4] often from multiple metastatic sites, which may have limited feasibility due to technical challenges or financial burden. Sequencing from blood plasma, however, has the potential to identify these changes without the invasiveness associated with solid tumor biopsies.[5–7]

Following the detection of mutant forms of *KRAS* and *NRAS* in the plasma of cancer patients, researchers have pursued cfDNA as a form of “liquid biopsy” of an individual’s cancer, using it to identify oncogenic alterations in a variety of malignancies.[8–14] Changes in circulating tumor DNA (ctDNA) over the course of treatment can be measured easily through serial sampling due to the minimally invasive nature of blood draws.[15–19] Previous studies have focused on quantifying ctDNA levels to measure disease burden,[15, 19, 20] searched for the emergence of resistance mutations to specific therapies,[18, 21–23] tracked tumor evolution,[18] and assessed prognosis[12, 24, 25] and recurrence risk.[16] The detection of ctDNA requires especially sensitive methods due to its dilution by the DNA from non-cancerous cells, with variant allele percentages as low as 0.01% in early disease.[12, 26, 27] The study of tumors of varying types and stages has found that while ctDNA levels vary significantly between samples, metastatic disease correlates with higher levels of cfDNA in the plasma and a higher fraction of ctDNA.[6, 28] The relative abundance of cfDNA and ctDNA makes it well-suited for whole-exome sequencing[18] which, unlike panels focusing on hotspot or patient-specific mutations, has the potential to identify novel mutations, giving it unique value in the study of therapeutic resistance and tumor evolution. Whole-exome sequencing from plasma has demonstrated high levels of concordance between mutations in the tumor tissue and cfDNA in metastatic disease; however, previously this has only been shown in samples with exceptionally high ctDNA levels (33–65% of cfDNA from tumor origin), greatly limiting its clinical utility.[18]

In this study, we investigated the feasibility of whole-exome sequencing from the plasma of two patients with metastatic disease. We found that with only minor alterations to our experimental and analytical methods we could accurately recapitulate the tumor genome from plasma, identify the same clinically relevant mutations identified by sequencing tumor biopsies, and gain novel information about the evolution of the disease. These methods were sensitive in a sample with an average ctDNA variant percentage of 3.7%, indicating approximately 7.4% of cfDNA was of tumor origin (ctDNA), sufficiently low to identify ctDNA for a substantial portion of metastatic patients.[6, 12, 16] We conclude that cfDNA sequencing of patients with metastatic cancer lends valuable insight to the study and treatment of the disease.

## Results

### Patient #1

**Fig 1:**
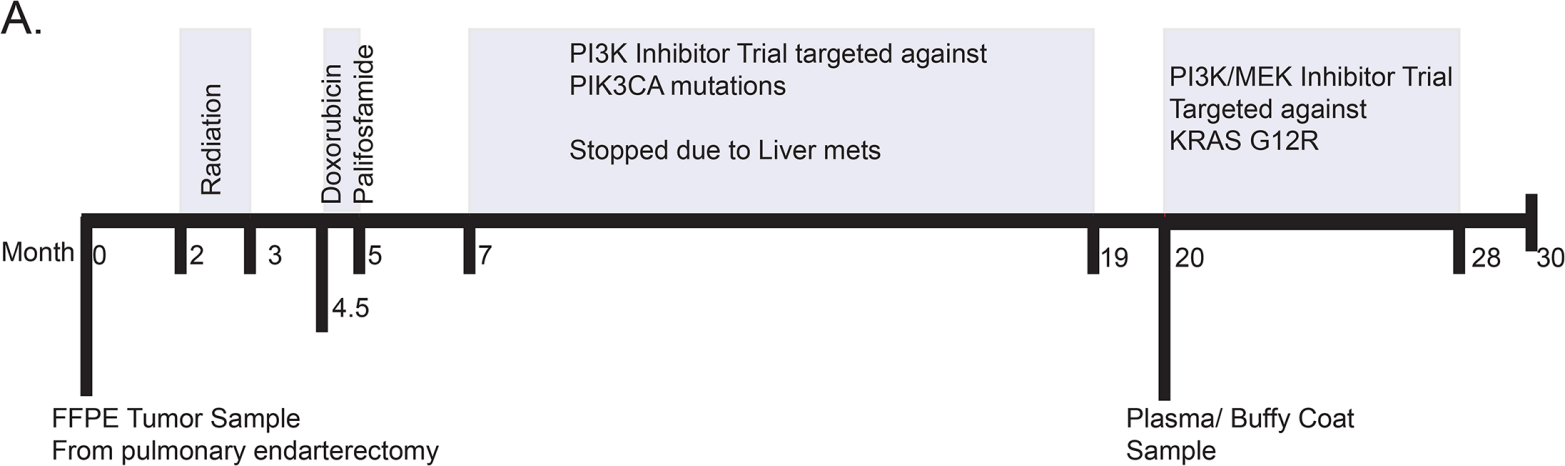
Overview of metastatic sarcoma patient treatment history. A) Patient diagnosed with intimal spindle cell sarcoma of the pulmonary artery. Treatments and sample collection indicated in months.

A 52-year-old female was diagnosed with primary intimal sarcoma of the pulmonary artery that was unresectable at presentation. The patient was initially treated with radiation followed by chemotherapy (Fig 1) and at this time her tumor was screened for oncogenic mutations using a multiplexed mass spectroscopy-based assay that revealed the presence of *PIK3CA* R88Q and Q546R in the primary tumor.[29] As a result, she entered a phase I clinical trial of a PI3 kinase inhibitor and had a partial response that lasted 12 months. Twenty months after diagnosis the primary tumor DNA was screened again using a targeted panel of an Ion Torrent PGM. This confirmed the *PIK3CA* mutations but also revealed *KRAS* G12R. A blood draw was taken at this time, isolating 1 ml of buffy coat and 25mls of plasma (Table 1). At the time of blood collection the patient had numerous lesions in the lungs, pulmonary artery, and liver (Table 1). Due to the high concentration of cfDNA in the plasma (63ng/ml), whole-exome sequencing was conducted. Based on the *KRAS* mutation, the patient was then enrolled in a phase Ib clinical trial combining MEK and PI3 kinase inhibitors. The treatment was stopped after eight months due to complications resulting from treatment, and the patient died 30 months after the initial diagnosis.

**Table 1:** Plasma Collection Summary. Volume of plasma collected from single blood draw. cfDNA quantified using Quan-iT HS pico green kit.

Sample	Primary Cancer Type	Volume Plasma Collected (ml)	Volume Plasma Extracted (ml)	cfDNA Concentration (ng/ml plasma)	Total cfDNA Extracted (ng)	Tumor Burden at Time of Collection
Patient #1	Sarcoma	25	25	63	1575	>6 chest lesions (0.5–2.6cm), 2 Liver lesions 1.3cm
Patient #2	Breast Cancer	15	10	98	980	>5 liver lesions (0.6–4.0cm), thoracic lesion in T11

**Fig 2:**
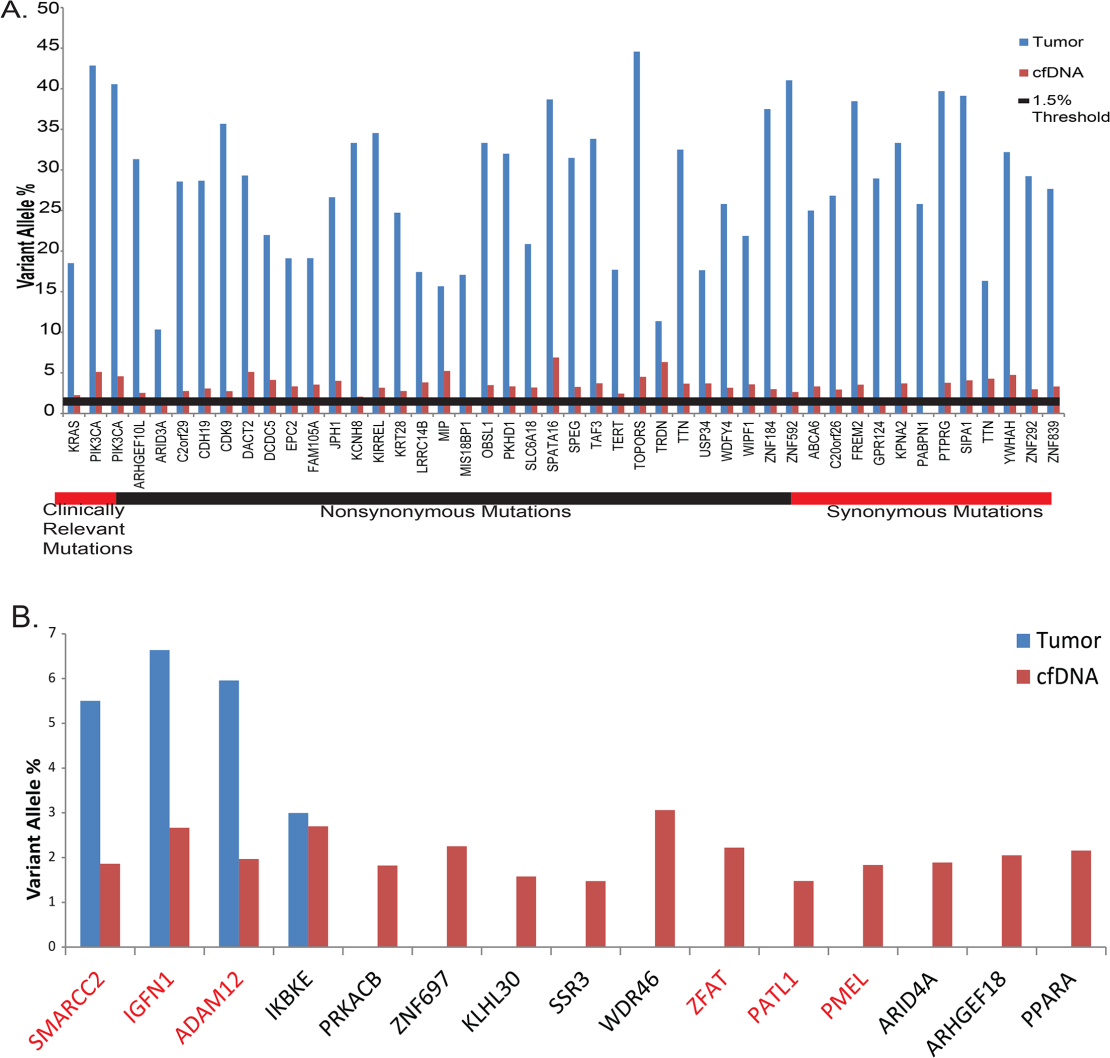
Patient #1 mutation calls and validation. A) Using a cutoff of 1.5% variant allele percentage, 46 of the 47 mutations present in the tumor were identified in the cfDNA. Estimating from the average variant allele percentage of 3.8%, 7.5% of the cfDNA was derived from the tumor. B) Fifteen additional mutations were called in the cfDNA which were not called in the tumor sample. Four of these are present in the tumor, but below our calling cutoff of 10% for the tumor. Genes highlighted in red were successfully validated via sequencing on the Ion Torrent PGM. Approximately 4,000 genomes of cfDNA were used as input to the validations, giving us a lower sensitivity bound of 0.025–0.5% depending on the site-specific background error rate.

Whole-exome sequencing of the primary formalin-fixed paraffin-embedded (FFPE) tumor revealed 48 somatic, exonic mutations (Fig 2A, Table 2. We conducted whole-exome sequencing of the cfDNA (524X average depth) and with a threshold of 1.5% variant allele percentage we identified 47 of the 48 somatic mutations present in the primary. At those 48 sites the mean sequencing depth in the cfDNA was 561X (181–1,197X). The average variant allele percentage across these 47 mutations was 3.7%, indicating that approximately 7.4% of the plasma DNA was of tumor origin. Importantly, we identified from plasma the activating *KRAS* G12R mutation and both activating mutations in *PIK3CA* (R88Q and Q546R). Controlling for sequencing depth, number of cfDNA mutant reads, or variant allele percentage in the primary tissue did not significantly improve the correlation.

**Table 2:** Sequencing Statistics. Summary of sequencing info for all ten sequencing runs. All reads are listed in millions. Accession numbers for.bam files uploaded to European Nucleotide Archive provided, for sarcoma patient all 3 cfDNA runs were combined in a single.bam file separated by read group.

Cancer Type	Tissue	ENA Accession Number	Input DNA (ng)	Reads (Millions)	Mapped Reads (%)	Paired Reads (%)	On Target Mapped Reads (%)	PCR Duplicates	Mean Sequencing Depth
Sarcoma	Buffy Coat	ERS700862	2000	392	361 (92.3)	342 (87.4)	210 (58)	29%	226
Sarcoma	Primary Tumor	ERS700863	500	116	114 (98.3)	113 (96.9)	93 (81.6)	31%	118
Sarcoma	cfDNA 1	ERS700864	750	250	243 (97.1)	204 (81.6)	143 (58.9)	34%	160
Sarcoma	cfDNA 2	ERS700864	110	155	154.8 (99.7)	147 (94.4)	124 (80.4)	12%	162
Sarcoma	cfDNA 3	ERS700864	110	186	185 (99.7)	175 (94.5)	155 (84.0)	16%	203
Sarcoma	Pooled cfDNA 1–3	ERS700864	970	591	583 (98.62)	526 (89.0)	423 (72.6)	n/a	524
Breast Cancer	Buffy Coat	ERS700858	412	182	181 (99.6)	180 (98.8)	154 (84.8)	20%	201
Breast Cancer	Primary Tumor	ERS700859	301	112	110.8 (99.2)	99.1 (88.8)	92 (82.8)	52%	118
Breast Cancer	Metastasis	ERS700860	341	173	171.8 (99.5)	170 (98.6)	140 (81.6)	22%	183
Breast Cancer	cfDNA	ERS700861	155	286	284.8 (99.5)	253 (88.5)	239 (83.8)	37%	309

**Fig 3:**
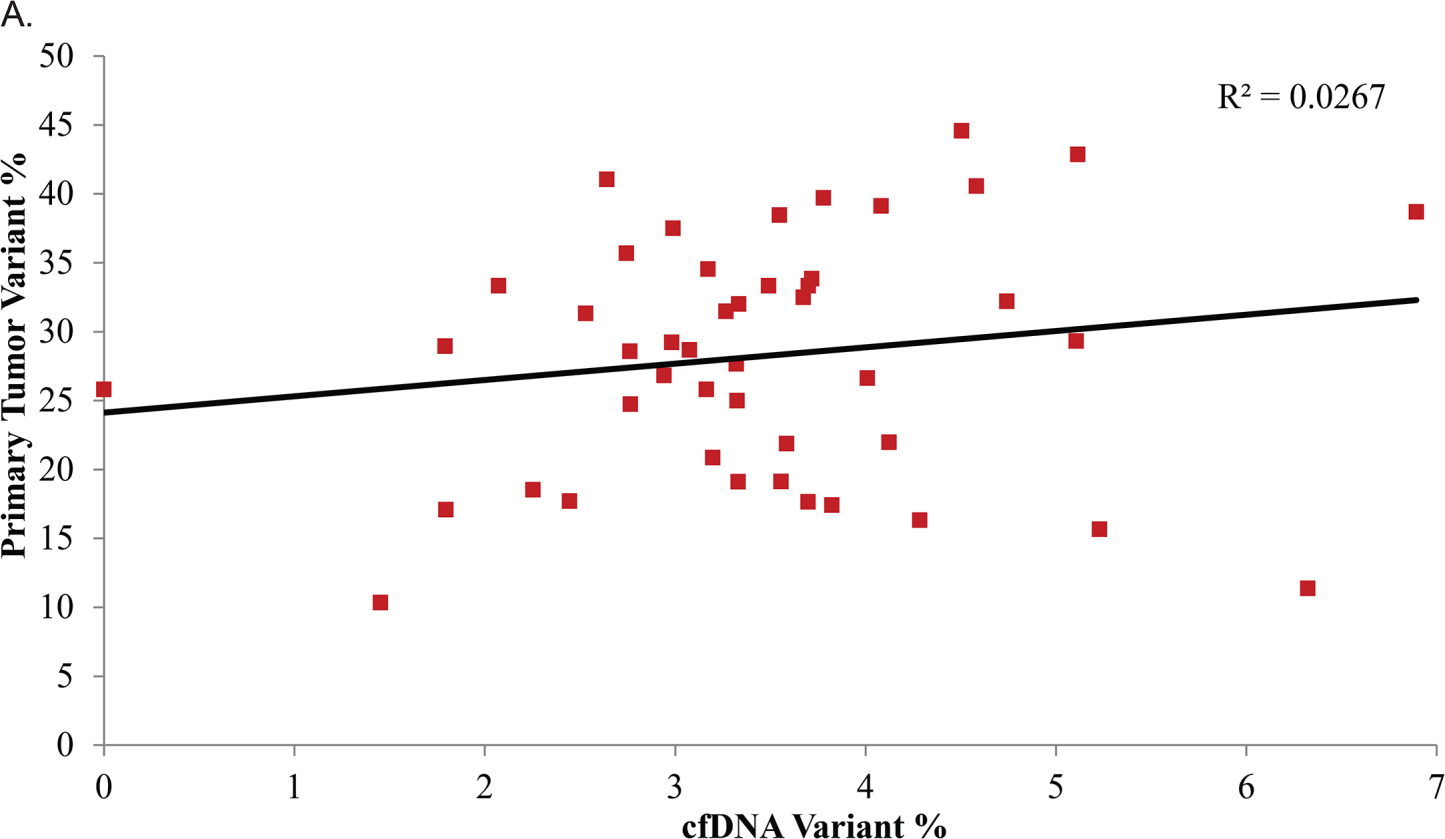
Variant allele percentage correlation. A) cfDNA variant allele percentage is poorly correlated with Primary tumor variant allele percentage.

Fifteen additional mutations were identified in the cfDNA. Among these, 11 were not present in the primary number and four were present in the primary tumor (Fig 2B), but at allele frequencies below our 10% threshold for calling them in the primary tumor. These mutations were chosen for validation by sequencing on the Ion Torrent PGM where six of them were confirmed, eight failed to validate, and one did not sequence (Fig 2B). The validation rate of 43% highlights the necessity of using orthologous sequencing methods in confirming the presence of low frequency mutations in cfDNA. cfDNA variant allele percentage correlated poorly with the primary tumor (Fig 3).

### Patient #2

**Fig 4:**
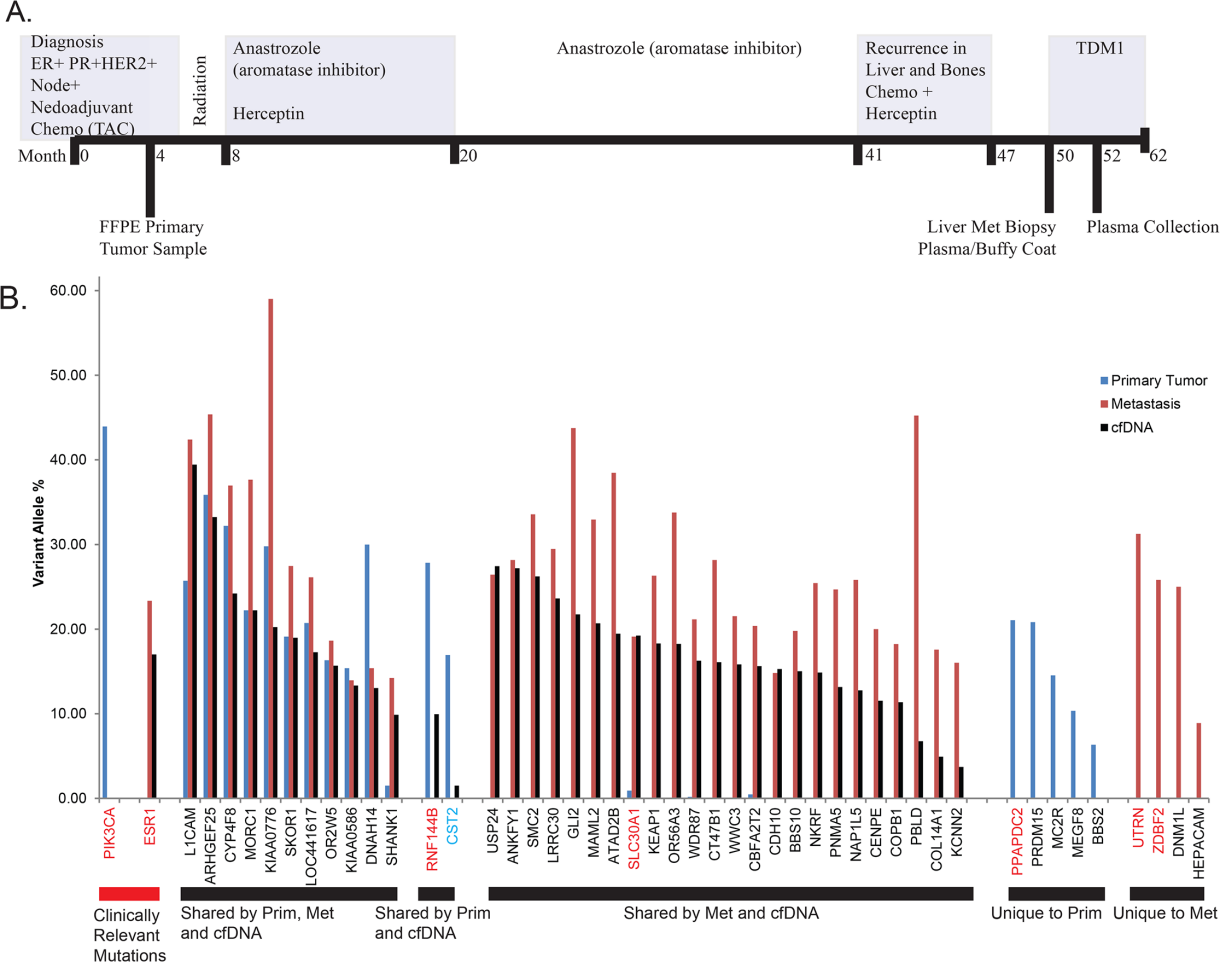
Patient #2 diagnosed with ER+/PR+/HER2+/Node+ breast carcinoma. A) Treatments and sample collection indicated in months. B) 48 total somatic mutations were called in the primary breast tumor and/or liver metastasis. 38 mutations were called in the cfDNA using a variant allele percentage cutoff of 1.5%. Genes in red were successfully validated on the Ion Torrent PGM, genes in blue failed to validate, genes were black were not validated.

A 41-year-old female was diagnosed with ER+ HER2+ breast cancer, which had spread to the lymph nodes. The patient underwent neoadjuvant chemotherapy (TAC) followed by a bilateral mastectomy and oophorectomy (Fig 4A). Following surgery, the patient underwent radiation therapy and was treated with Trastuzumab for one year and Anastrozole for 33 months, until the discovery of a 4cm liver lesion and bone metastases at the 11^th^ thoracic vertebra (T11). Additional chemotherapy and Herceptin were administered but the treatment was stopped following identification of liver metastases. At this time we collected a blood draw approximately 30 minutes before a liver biopsy was taken and obtained an archived FFPE sample of the primary tumor. The blood draw yielded 15mls of plasma at an average cfDNA concentration of 98ng/ml (Table 1). Following the first plasma sample the patient underwent treatment with the anti-Her2 drug TDM1 but following an initial partial response died 62 months after initial diagnosis.

**Fig 5:**
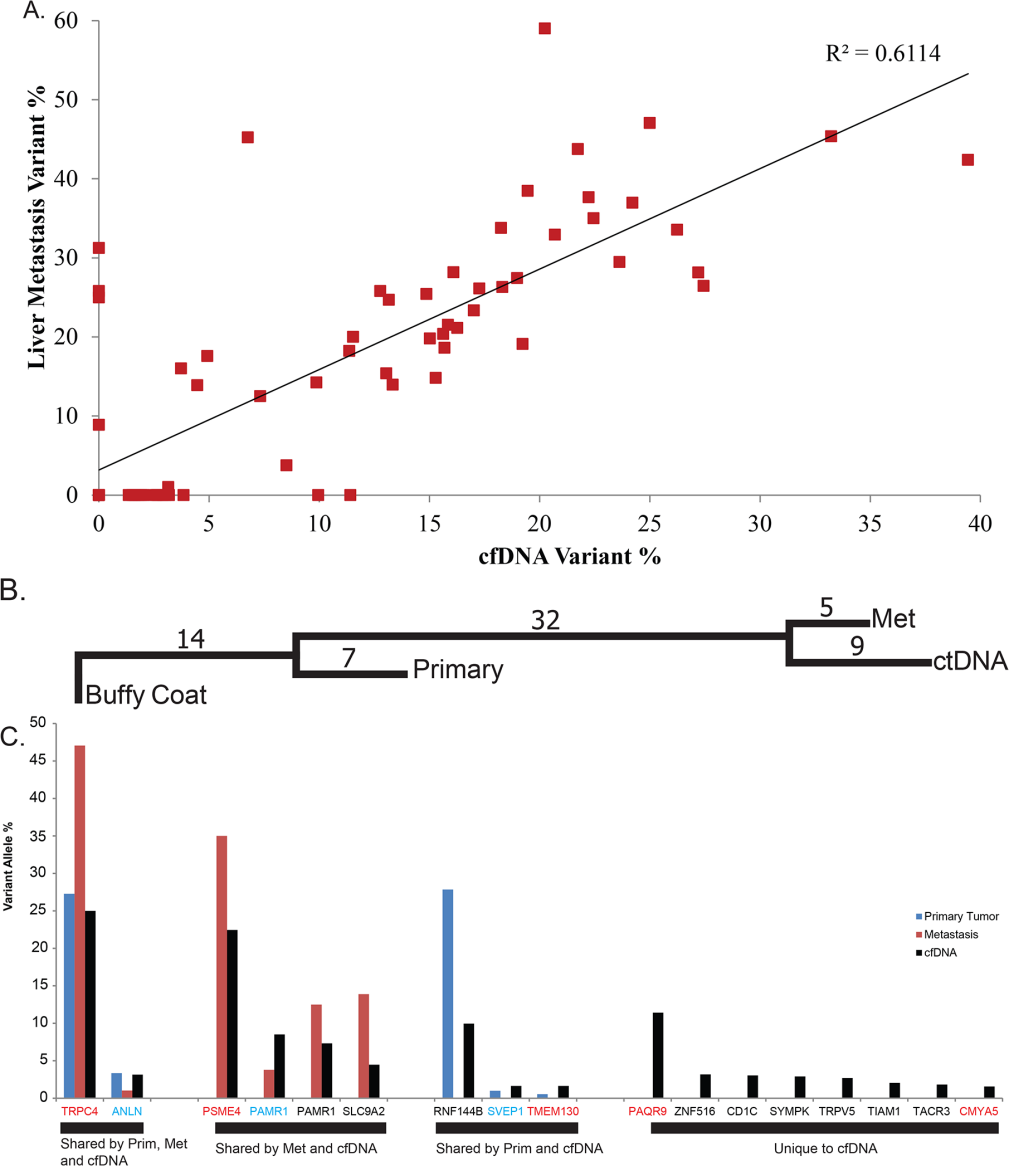
cfDNA and liver metastasis DNA are well correlated. A) cfDNA variant allele percentage is correlated with liver metastasis variant allele percentage B) Maximum parsimony tree showing relatedness of samples, branch length are number of somatic, nonsynonymous mutations C) Seventeen additional mutations were identified uniquely in cfDNA, 9 of which have reads supporting them in the primary and/or met, but where not called due to insufficient sequencing depth or variant allele percentage. Genes in red were successfully validated on the Ion Torrent PGM, genes in blue failed to validate, genes were black were not validated.

Whole-exome sequencing of the primary tumor and liver metastasis revealed a total of 48 nonsynonymous somatic mutations (Fig 4B, Table 2). Sequencing of cfDNA to an average depth of 309X identified 38 of these mutations with an average variant allele percentage of 14%, indicating approximately 28% of cfDNA was of tumor origin. cfDNA VAP correlated well with the VAP in the liver metastasis (Fig 5A and 5B), but correlated poorly with the primary tumor (data not shown). Additional deep sequencing confirmed that an activating *PIK3CA* (H1047R) mutation was present only in the primary tumor, not in the liver metastasis or cfDNA, indicating that either the mutation emerged after metastasis, or was not present in the subpopulation that seeded the metastasis. Seventeen additional somatic nonsynonymous mutations were called from the plasma sample. Closer examination revealed that eight of these (47%) were unique to the plasma, potentially originating from metastatic sites not sampled (Fig 5C). Two of those mutations were selected for validation via Ion Torrent PGM, both of them successfully validated (Fig 5C).

**Fig 6:**
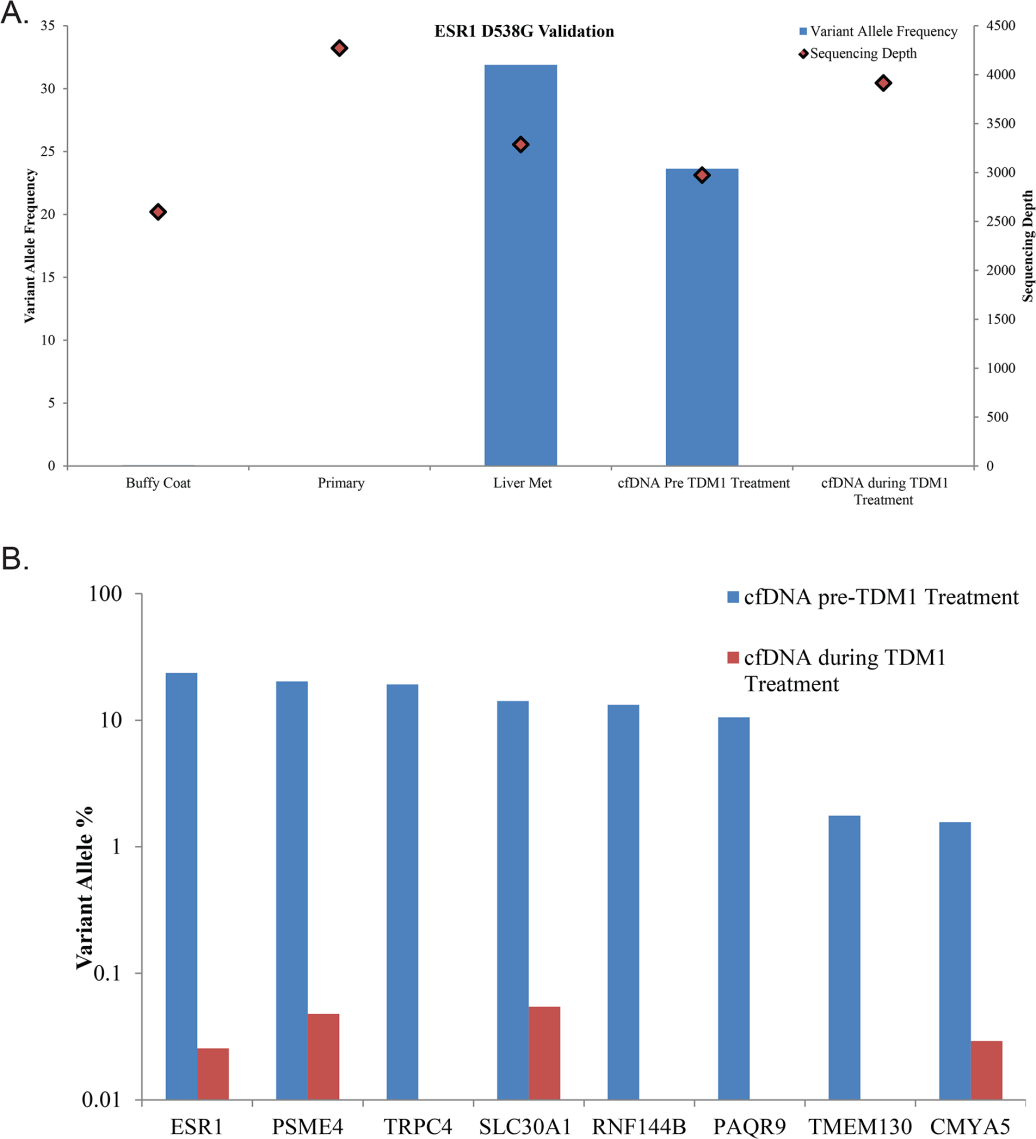
Patient #2 targeted resequencing. A) The *ESR1* mutation was sequenced to greater depth on the Ion Torrent PGM. B) Comparison of allele frequencies between pre- and during-TDM1 treatment cfDNA samples for eight mutations present in the pre-treatment sample.

By sequencing cfDNA from plasma we are able to get a snapshot of the tumor, likely from multiple metastatic sites. Here, the high correlation between the liver metastasis and cfDNA indicates that considerable information about the current tumor genome could be gained without the need for a biopsy. A mutation in *ESR1* (D538G), which has been shown to impart resistance to estrogen deprivation therapy, was found in both biopsies of the metastases and the cfDNA.[30, 31] This mutation was not present in the initial exome sequence of the primary tumor and its absence was confirmed by subsequent validation sequencing of *ESR1* to a depth of 4,272X (Fig 6A). It is likely that the resistance of the tumor to the aromatase inhibitor Anastrozole can be explained by the mutant *ESR1*. This mutation was confirmed in a CLIA laboratory and anti-Estrogen Receptor treatments were considered between cfDNA sequencing and patient death. A total of 15 mutations were selected for validation on the Ion Torrent PGM, 13 of which were validated (Figs 4B and 5C). A second plasma sample was taken during response to TDM1 treatment (as determined by CT scan) and eight mutations present in the pre-treatment cfDNA sample were quantified in the during-treatment sample (Fig 6B). The pre-treatment cfDNA sample had a mean variant allele percentage of 13% across these eight sites while the during-treatment sample had a mean variant allele percentage of only 0.04% in the four sites containing mutant reads and no detectable mutant reads in four of the mutations tested.

## Discussion

In this study, we have demonstrated that whole-exome sequencing of cfDNA from patients with metastatic cancer can accurately identify clinically actionable mutations, and requires only minimal alterations to well-established sequencing protocols. We were able to sequence and gain valuable data from a plasma sample with a mean variant allele percentage of 3.7%, much lower than values demonstrated in previous studies and well below the frequencies of a substantial portion of metastatic cancer patients.[12, 15, 16, 18, 19] Adoption of this approach has the potential to greatly expand the utility of sequencing versus the biopsy-dependent approaches which are currently the standard of care. Mutations present in the cfDNA tightly correlated with mutations present in a synchronous metastasis sample, indicating that sequencing cfDNA can generate a more accurate picture of a patient’s metastatic tumor genome than relying on a biopsy of the primary tumor. The cfDNA tightly correlates with tumor tissue taken at the time of plasma acquisition and can therefore be used to take “snapshots” of the cancer genome. Additionally, mutations unique to cfDNA were found in both patients, potentially representing lesions not sampled by biopsy. Validation via orthologous sequencing methods confirmed that these mutations were not from normal tissue or the result of sequencing errors and were likely from sites not present in the biopsy. The inability to sample all metastatic sites within a cancer patient is a severe limitation of current sequencing techniques, and may be resolved with minimal modifications to standard sequencing procedures using cfDNA.

The two patients in this study had high levels of cfDNA in their plasma (Table 1), which allowed us to use over 100ng of cfDNA to construct our sequencing libraries. However, for many patients a concentration of 10ng of cfDNA per ml of plasma is more typical, indicating that multiple blood draws are required to get sufficient material for sequencing. Realizing this, we adopted the methods outlined in the Capp-Seq paper from the Diehn lab [19] that allows libraries to be made more efficiently, requiring less initial input DNA. Using these methods we successfully produced complex libraries from less than 40ng of cfDNA and successfully sequenced ~25% of the input DNA molecules (opposed to the ~1% efficiency achieved in our study). This improvement has allowed us to sequence sufficient cfDNA for nearly all our subjects.

Another advantage of sequencing cfDNA is the ability to sequence serially-collected and minimally-invasive plasma samples, allowing for near real-time monitoring of the tumor genome during treatment. The identification of emerging mutations may allow therapies to be started or stopped as soon as the tumor environment renders this advantageous. In the case of patient #2, it is possible that serial cfDNA sequencing would have identified the emergence of the *ESR1* mutation and treatment may have been adjusted from estrogen deprivation therapy (Anastrozole) to one targeting the estrogen receptor itself (*e*.*g*. Fulvestrant): this shift, and potentially others, may have delayed the progression of disease. In addition to looking for known resistance mechanisms, the nature of whole-exome sequencing allows for the identification of novel recurrent resistance mechanisms in a cohort of patients undergoing the same treatment, which may not be included in a targeted panel. Notably, during the response of patient #2 to TDM1 there was a dramatic reduction in the level of ctDNA, rendering it nearly undetectable by our sequencing approach. Monitoring via exome sequence during such periods would require extremely high sequencing depth, which would be prohibitively expensive with current sequencing costs.

A substantial focus has been placed on the sequencing of primary tumors and massive sequencing projects (TCGA *et al*.*)* have revealed a considerable amount of information about driver mutations in a variety of cancers. However, metastatic tumors, which are responsible for most patient deaths, are comparatively understudied. By sequencing primary tumors along with serially collected plasma samples it is possible to monitor metastatic progression at a genomic level. In patient #2 we observed an activating *PIK3CA* mutation in the primary tumor that was not seen in either the liver metastasis or cfDNA. It is likely that either the *PIK3CA* mutation became clonal after the metastatic process or that the mutation was not present in the metastatic clone; regardless, treatment with a PI3K inhibitor may have been effective in shrinking the primary lesion, but would have been ineffective against any of the distant metastasis. In contrast, sequencing of patient #1 showed that the cfDNA shared contained nearly all of the mutations identified in the primary tumor. While we were unable to get a sample of the metastasis, the low number of mutations unique to the cfDNA means it is not unreasonable to infer that there were relatively few differences between the metastasis and primary tumor. Sequencing cfDNA from larger cohort of patients may help us understand how metastatic progression varies in different tumor types and may identify therapeutically relevant patterns. The clinical utility of this method will depend largely on the systematic assignment of targeted therapies to identified cfDNA mutations.

Notably, services for cfDNA sequencing are becoming commercially available, but are based on panels and therefore have limited utility in a research setting. We demonstrate here that there is significant value of whole-exome sequencing from cfDNA.

## Materials and Methods

### Patient enrollment

Written consent was obtained from two patients with metastatic cancer for enrollment in this study. The study and consent procedures were approved by the Oregon Health & Science University Institutional Review Board and in accordance with federal and institutional guidelines. Up to 40mls of blood was collected in EDTA tubes. Plasma was isolated as described previously [16] and stored at -80°C until cfDNA was extracted using the QIAamp Circulating Nucleic Acid kit (Qiagen). Buffy coat was isolated from the same blood sample and DNA was extracted using the DNA Blood Mini kit (Qiagen). As part of the aforementioned study and consent procedure, FFPE tissue from the patient’s primary tumors was acquired from archived pathology samples. Patient #1’s sample was acquired from the University of Washington Pathology Department in Seattle, WA (http://www.pathology.washington.edu/clinical/dermpath/contactinfo). Patient #2’s sample was acquired from Compass Oncology in Vancouver, Washington (http://compassoncology.com). FFPE tissue was extracted using the DNA FFPE Tissue kit (Qiagen). The same patient’s liver metastasis was taken from a frozen core biopsy and extracted with the DNeasy Blood & Tissue kit (Qiagen).

### Whole-exome sequencing

A minimum of 100ng of cfDNA and 0.3–2μg of DNA from buffy coat and tumor tissue were used to create sequencing libraries. Agilent SureSelect XT reagents and protocol were used to prepare sequencing libraries. DNA from buffy coat and tumor tissue was sonicated to an average size of 150bp using a Covaris E220. Plasma DNA samples were not sonicated, as plasma DNA is already highly fragmented. Hybrid capture was conducted using Agilent SureSelectXT Human All Exon V4+UTRs. 100bp paired-end sequencing was conducted on an Illumina HiSeq 2000. An entire lane was dedicated to sequencing plasma DNA samples and all other libraries were sequenced two-to-a-lane. To maximize sequencing depth and avoid PCR duplicates, the plasma sample from the patient with metastatic sarcoma was made into three separate libraries, each sequenced on one full lane each, giving an average sequencing depth of 1,034X. Only a single library was needed to achieve sufficient coverage of cfDNA for the patient #2.

### Bioinformatic analysis

In order to detect mutations we aligned HiSeq paired-end reads with hg19 human reference genome using bwa.[32] We used bwa aln to find the coordinates of input reads and then used bwa mem in order to generate alignments in a sam format. We converted the sam format to bam (binary) format using Samtools import. After sorting and indexing the reads in the bam formatted file, we use Picard Tools[33] MarkDuplicates to remove duplicate reads generated during the PCR amplification stage: removal is done by finding all reads that have identical 5’ coordinates and keeping only the read pair with the highest base quality sums. After duplicate removal we realigned reads around SNVs and indels using the GATK Software Library.[34, 35] The three libraries of the sarcoma patient were combined after PCR duplicate removal: local positions to target for realignment were called using RealignerTargetCreator and the reads were realigned using IndelRealigner. Finally, quality scores were recalibrated. This was done using GATK BaseRecalibrator and PrintReads, which binned reads based on the original quality score, the dinucleotide, and the position within the read. Sequencing statistics are summarized in Table 2 and were generated using Samtools flagstat, GAKT DepthOfCoverage, and Bedtools pairToBed.

To call mutations we compared the tumor samples with the normal samples using muTect v1.1.4 using the buffy coat as a matched normal.[36] Variants were considered somatic mutations if: (a) they were not present in the dbSNP database (except if the variant was also in the COSMIC database eg *KRAS* and *PIK3CA* mutations), (b) there was ≥30x sequencing depth at that site in the tumor/plasma sample and ≥10x sequencing depth in the matched normal sample, (c) it had a variant allele percentage of ≥10% for the tumor samples and ≥1.5% for plasma samples, and (d) there were at least two reads containing the variant allele. Mutations in cfDNA were then further filtered out if the matched normal had >1 read supporting the mutation or the mutation was only present in one strand of the cfDNA. Impact of variants was checked using Mutation Assessor v2 (www.mutationassessor.org).

### Mutation validation

Primers were designed to cover a selection of mutations identified in each patient and then used to PCR amplify buffy coat, plasma, and tumor DNA samples from both patients. For each sample, amplicons were pooled in equimolar amounts and 10–100 ng were used for library creation using the Ion Xpress Plus Fragment Library Kit. Sequencing templates were generated using emulsion PCR on the Ion OneTouch 2 using the Ion PGM Template OT2 200 kit. Up to six barcoded samples were multiplexed on Ion 316 v2 chips. Sequencing was performed on a Personal Genome Machine (PGM) sequencer (Ion Torrent) using the Ion PGM 200 v2 sequencing kit. Torrent Suite software version 4.0.2 was employed to align reads to hg19. Reads were visualized using IGV v 2.2.32 (Broad Institute) and variant allele frequencies were determined for sites previously identified via Illumina sequencing.
